# Recent advances in protein extraction methods from bone tissue for proteomics analysis

**DOI:** 10.1098/rsos.261692

**Published:** 2026-10-14

**Authors:** Karoline B. do. Nascimento, Bruno L. Graciliano Silva, Felipe E. Pinotti, Julio Cesar Sánchez-Puetate, Morgana R. Guimarães-Stabili, Camila C. Marcantonio, Rosemary Adriana C. Marcantonio

**Affiliations:** ^1^ Universidade Estadual Paulista Julio de Mesquita Filho, Araraquara, São Paulo, Brazil; ^2^ Pontifica Universidade Católica de Campinas, Campinas, São Paulo, Brazil; ^3^ Universidad San Francisco de Quito, Quito, Pichincha, Ecuador

**Keywords:** bone proteomics, protein extraction, mass spectrometry

## Abstract

Proteomic analysis is a fundamental tool for obtaining new information about biological functions that occur at the molecular level in the body, allowing the understanding of physiological and pathological processes, as well as contributing to the diagnosis and prevention of diseases. However, a critical step for the success of these analyses is the protein extraction from the tissue of interest, with well-described and reproducible protocols still scarce in the current literature, especially for bone tissues. Therefore, the main objective of this study was to present a reliable and standardized methodology for protein extraction from hard tissue samples. The methodology employed was based on an extraction protocol using demineralized bone tissue, with more detailed experimental parameters such as incubation times, temperatures and sample storage conditions. As a result, 1429 proteins previously described for the species *Rattus norvegicus* were identified, along with 138 uncharacterized proteins and the detection of signalling pathways strongly associated with bone metabolism. The proposed protocol has the potential for adaptation to different experimental contexts, being adjustable for various species and types of bone, considering variations in density, degree of mineralization and structural characteristics of the analysed tissue.

## 1. Introduction

Proteomic analysis is an essential tool for investigating biological functions in physiological and pathological conditions [1]. By allowing the identification, quantification and structural characterization of proteins in different tissues, proteomics provides crucial information about processes that regulate homoeostasis, disease development and response to treatments [2,3]. Through this approach, it is possible to map protein profiles and detect functional alterations associated with pathological states, contributing to early diagnosis, therapeutic monitoring and identification of potential biomarkers [4,5]. Furthermore, proteomics has been increasingly applied in areas such as cancer research, immunology, infectious diseases, pharmacology and forensic sciences, demonstrating its impact and versatility in the biomedical field [6].

Various techniques can be employed for proteomic analysis, with two of the most commonly used being two-dimensional polyacrylamide gel electrophoresis (2D-PAGE) and mass spectrometry (MS). MS, in particular, enables qualitative and quantitative analysis of proteins, as well as their identification through comparison with existing databases [7–9]. Currently, two approaches stand out: ‘top-down’ analyses, where intact proteins are directly examined, and ‘bottom-up’ analyses, which involve prior enzymatic digestion and subsequent identification of the resulting peptides. These advancements have significantly expanded the understanding of protein–protein interaction (PPI) networks and contributed to more refined studies of molecular mechanisms, especially when combined with fractionation techniques, nanoESI chromatography and advanced database search algorithms [9].

However, to obtain reliable proteomic information, proper sample preparation is a critical step [10]. The efficiency of protein extraction directly influences the quality of the generated data, as losses during extraction or protein degradation can compromise subsequent analysis [9]. While protocols are often described for soft tissues, there are still significant methodological limitations when it comes to mineralized tissues, such as bone tissue [11]. Preserving protein integrity, storage and transportation conditions, and the correct choice of buffers and denaturing agents are crucial factors to minimize losses and ensure the representativeness of the obtained protein profiles.

Existing literature on protein extraction from bone tissue reveals both the potential of this biological source and the technical challenges associated with it. Studies like those of Bell *et al.* [11], using human alveolar bone, and Goldberg *et al.* [12], pioneers in extraction from demineralization protocols, demonstrate the feasibility of bone proteomic analysis. However, such methodologies often require adaptations, prolonged steps or specific experimental conditions that hinder their reproducibility. Jiang *et al.* [13] compared different extraction approaches using canine bone tissue under three distinct conditions: demineralized, mineralized and crushed, demonstrating that prior demineralization can significantly increase protein yield. More recent studies, such as those by Cleland & Vashishth [14] and Licini *et al.* [15], reinforce that there is no consensus on critical parameters, including the ideal concentration of demineralizing agents, incubation time, temperature and interlaboratory repeatability. Thus, the availability of complete, standardized and detailed protocols that allow for broad application in animal and human models is still limited.

Given this gap, there is a clear need for reproducible and clearly described methodologies that can be widely applied for protein extraction from bone samples with efficiency and consistency. This study aims to establish a standardized protocol for protein extraction from bone tissue, focusing on optimizing the demineralization step and using nano liquid chromatography coupled with tandem MS (LC–MS/MS). By presenting a clear, detailed and tested methodology, it is expected to contribute to the standardization of laboratory procedures, promote reproducibility among studies and expand the use of bone tissue as a source for proteomic investigations.

## 2. Material and methods

### 2.1. Protein extraction

Thirty-six samples of tibia bone tissue (approx. 3 × 3 mm^2^) from male rats (*Rattus norvegicus* var. *albinus*, Holtzman strain) were used. The animals were kept under controlled environmental and feeding conditions, and the collection was performed immediately after euthanasia, according to the protocol approved by the Ethics Committee on Animal Use of the Araraquara School of Dentistry/UNESP (07/2019). After removal, each sample was individually placed in sterile microtubes (1.5 ml) and stored at −80°C until the protein processing step.

Mechanical removal of residual soft tissue was performed with a sterile periodontal curette (Gracey 5-6, Golgran, São Caetano do Sul, Brazil). The bones were kept intact throughout this procedure; they were not fragmented prior to demineralization, and the epiphyses were not removed.

Subsequently, 400 µl of protease inhibitor cocktail (Santa Cruz Biotechnology, no. J0606) was added to each microtube, keeping the samples at 4°C overnight. After centrifugation (10 000 × *g*, 10 min, 4°C), the supernatant was discarded, and initial demineralization was carried out by adding 400 µl of 1.2 M HCl (pH < 1), kept at 4°C overnight. The supernatant generated after centrifugation (10 000 × *g*, 10 min, 4°C) was removed.

Next, the samples were washed three times with ultra-pure water (Milli-*Q*) and incubated for 72 h at 4°C in 400 µl of Buffer Solution 1 (50 µl of protease inhibitor cocktail + 175 µl of guanidine-HCl (Promega, lot no. 0000422160) 6 M + 175 µl of Tris (BIO-RAD, lot no. 1610716) 100 mM, pH 7.4). After centrifugation (10 000 × *g*, 10 min, 4°C), the supernatant was removed.

Subsequently, the samples were washed again with Milli-*Q* and incubated for 72 h at 4°C in 400 µL of Buffer Solution 2 (50 µl of protease inhibitor + 116.7 µl of Tris 100 mM + 116.7 µl of guanidine-HCl 6 M + 116.7 µl of tetrasodium EDTA (Êxodo Científica, lot no. 2103261866), pH 7.4). After extracting the supernatant, the samples were washed with ultra-pure water and then placed in a solution with 400 µl of 6 M HCl (overnight, 4°C) for the second demineralization step.

For lipid removal and precipitation, the samples were washed with ultra-pure water, incubated in 400 µl of 100% acetone (analytical) at −20°C overnight, centrifuged and the supernatant discarded. The final extraction step was carried out by incubating the samples for 72 h at −20°C in Buffer Solution 3 (400 µl of Tris 100 mM + guanidine-HCl 6 M, pH 8.1). The resulting supernatant after centrifugation was collected as the final extract, intended for protein quantification (figure 1).

**Figure 1.**
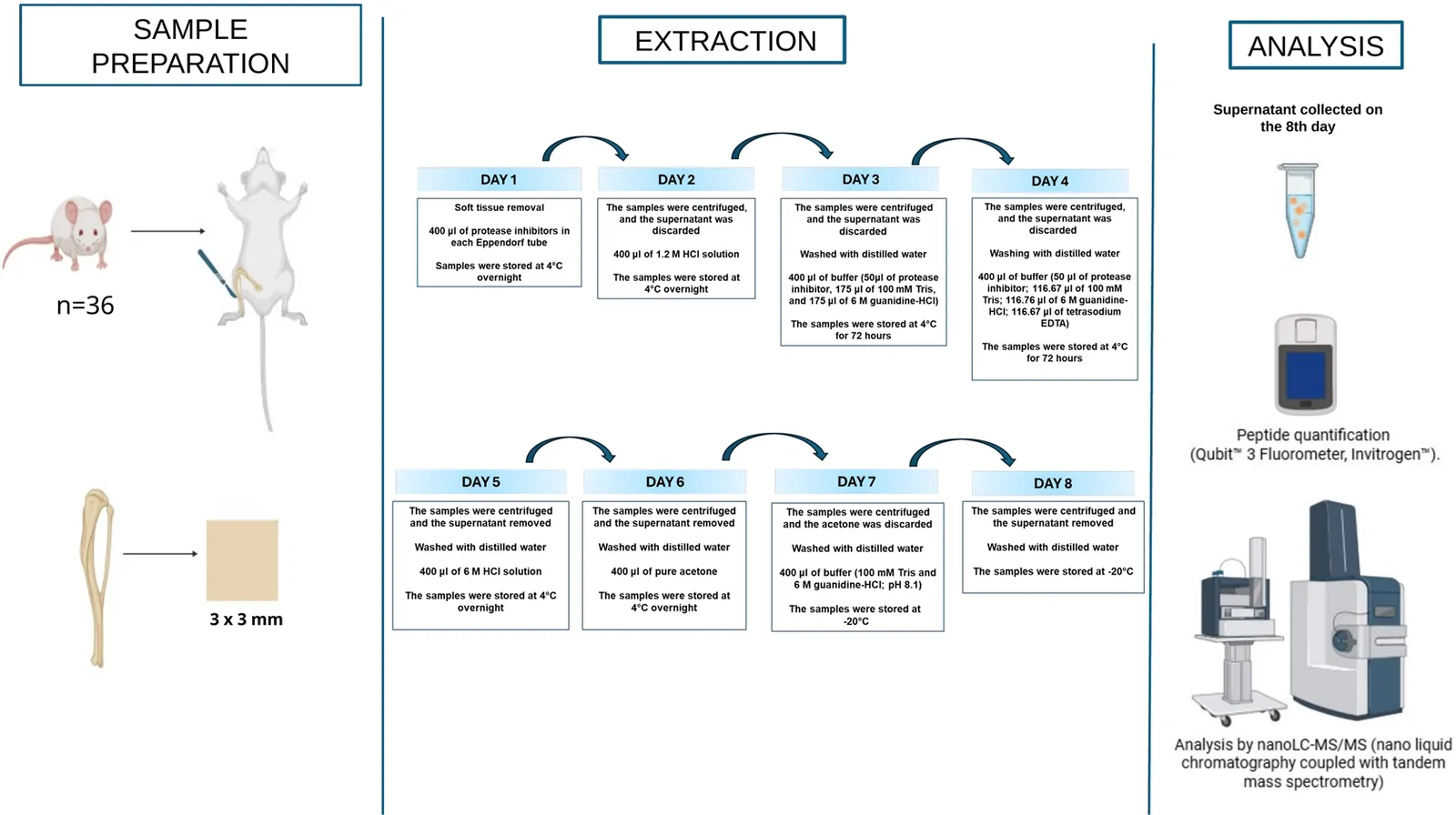
Experimental protocol scheme for protein extraction, modified from Jiang *et al*. [13].

### 2.2. Lyophilization

The final extract was subjected to lyophilization (FreeZone, Labconco) at −50°C, 0.05 mbar, until completely dried (approx. 24 h). Lyophilization was used for protein stabilization and prevention of proteolytic and oxidative degradation before digestion steps.

### 2.3. Tryptic digestion

Prior to tryptic digestion, the protein extracts were diluted ninefold with Milli-*Q* water to reduce the concentration of guanidine to a level compatible with trypsin activity (below 1 M), thereby avoiding inhibition of enzymatic digestion. No buffer exchange was performed before lyophilization.

The lyophilized samples were resuspended in 300 µl of Milli-*Q* water and quantified using the Qubit fluorometric method (Qubit 3 Fluorometer, Invitrogen). A total of 100 µg of protein was transferred per microtube. The pH was adjusted to 8.5 with 1 M ammonium bicarbonate when necessary.

Protein reduction was performed with DTT (10 mM final) at 90°C for 10 min followed by incubation at 37°C for 60 min. Alkylation was carried out with 40 mM iodoacetamide (IAA) for 30 min in a light-protected environment. Then, trypsin (Promega, sequencing grade modified trypsin) at 0.2 µg µl^−1^, enzyme: substrate ratio 1:50, was added and incubated overnight (16–20 h) at 37°C. The reaction was stopped by adding 10 µl of 1% formic acid.

### 2.4. Column preparation and sample clean-up

Purification was performed using manually packed C18 microcolumns with C18 discs (empire, R2, reverse phase) following a homemade solid-phase extraction protocol. The columns were conditioned with 100% acetonitrile (ACN) and equilibrated twice with 0.1% TFA. The samples were loaded, washed with two passes of 0.1% TFA and the peptides eluted with

— Elution 1: 50% ACN + 0.1% TFA
— Elution 2: 70% ACN + 0.1% TFA

The eluates were dried in a SpeedVac (Thermo Scientific) at 40°C for 2 h.

### 2.5. Peptide quantification

After drying, the samples were resuspended in 0.1% formic acid for subsequent peptide quantification using the Qubit protein quantification device (Qubit 3 Fluorometer, Invitrogen). After quantification, the concentration was adjusted to 0.5 or 0.2 µg µl^−1^ with 0.1% formic acid, and 4 or 10 µl (2 µg of protein) were used for injection into the mass spectrometer. The samples were transferred to injection vials and immediately analysed by nanoLC–MS/MS (nano liquid chromatography coupled with tandem mass spectrometry). EASY-nLC 1000 (Thermo Scientific) coupled to the Q Exactive Plus mass spectrometer (Thermo Scientific).

Peptide mixtures were analysed using an EASY-nLC 1000 system (Thermo Scientific, Bremen, Germany) coupled online to a Q Exactive Plus Orbitrap mass spectrometer (Thermo Scientific). Peptides were separated on a homemade analytical column (75 µm × 25 cm) packed with ReproSil Gold C18 resin (3 µm, 200 Å; Dr. Maisch) preceded by a homemade trap column (100 µm × 3 cm) packed with ReproSil C18 resin (5 µm, 200 A; Dr. Maisch). The mobile phase consisted of solvent A (95% water, 5% acetonitrile and 0.1% formic acid) and solvent B (95% acetonitrile, 5% water and 0.1% formic acid). Peptides were eluted at a flow rate of 250 nl min^−1^ using the following gradient: 5% B at 0 min, 23% B at 25 min, 45% B at 50 min, 95% B at 55 min and maintained at 95% B until 60 min.

The mass spectrometer was operated in data-dependent acquisition (DDA) mode. Full MS (MS1) scans were acquired in the Orbitrap at a resolution of 70 000 over an *m*/*z* range of 375–1650, with an AGC target of 3 × 10⁶ and a maximum injection time of 50 ms. The top 20 most intense precursor ions were selected for higher-energy collisional dissociation (HCD) using a normalized collision energy of 30, an isolation window of 1.4 *m*/*z* and dynamic exclusion of 40 s (1.2 *m*/*z* exclusion window). MS/MS spectra were acquired at a resolution of 17 500, with an AGC target of 1 × 10⁶, a maximum injection time of 100 ms, a scan range of 350–2000 *m*/*z* and a signal threshold of 1 × 10⁵. Unassigned ions and singly charged precursor ions were excluded from fragmentation.

### 2.6. Bioinformatic analysis

Mass processing was performed in Proteome Discoverer v.2.1 (Thermo Fisher) using the SEQUEST HT algorithm against the UniProt database (*R. norvegicus*, last updated 05/2024).

— Fixed modification carbamidomethylation (Cys)
— Variable modifications: oxidation (Met), deamidation (Asn) and acetylation (N-terminal)
— Maximum missed cleavages: 2
— Tolerance: precursor ions = 10 ppm | fragment ions = 0.02 Da
— FDR: <1% at protein, peptide and PSM levels
— Proteins grouped by maximum parsimony principle

Functional enrichment analysis was performed using Gene Ontology (GO) and Kyoto Encyclopedia of Genes and Genomes (KEGG) enrichment analysis with DAVID v.6.8. Protein abundance differences were determined by Student's *t*-test with Benjamini–Hochberg correction (*p* ≤ 0.05).

## 3. Results

Thirty-six bone block samples removed from rodent tibiae were used for proteomic analysis. Among the results obtained using the modified extraction protocol by Jiang *et al.* [13], 1429 known proteins for the species *R. norvegicus* and 138 unknown proteins were detected (supplementary material, table S1).

### 3.1. Enrichment analysis

In the GO enrichment analysis, performed using the DAVID software, the identified proteins were analysed according to cellular component (CC), biological process (BP) and molecular function (MF) terms (figure 2).

**Figure 2.**
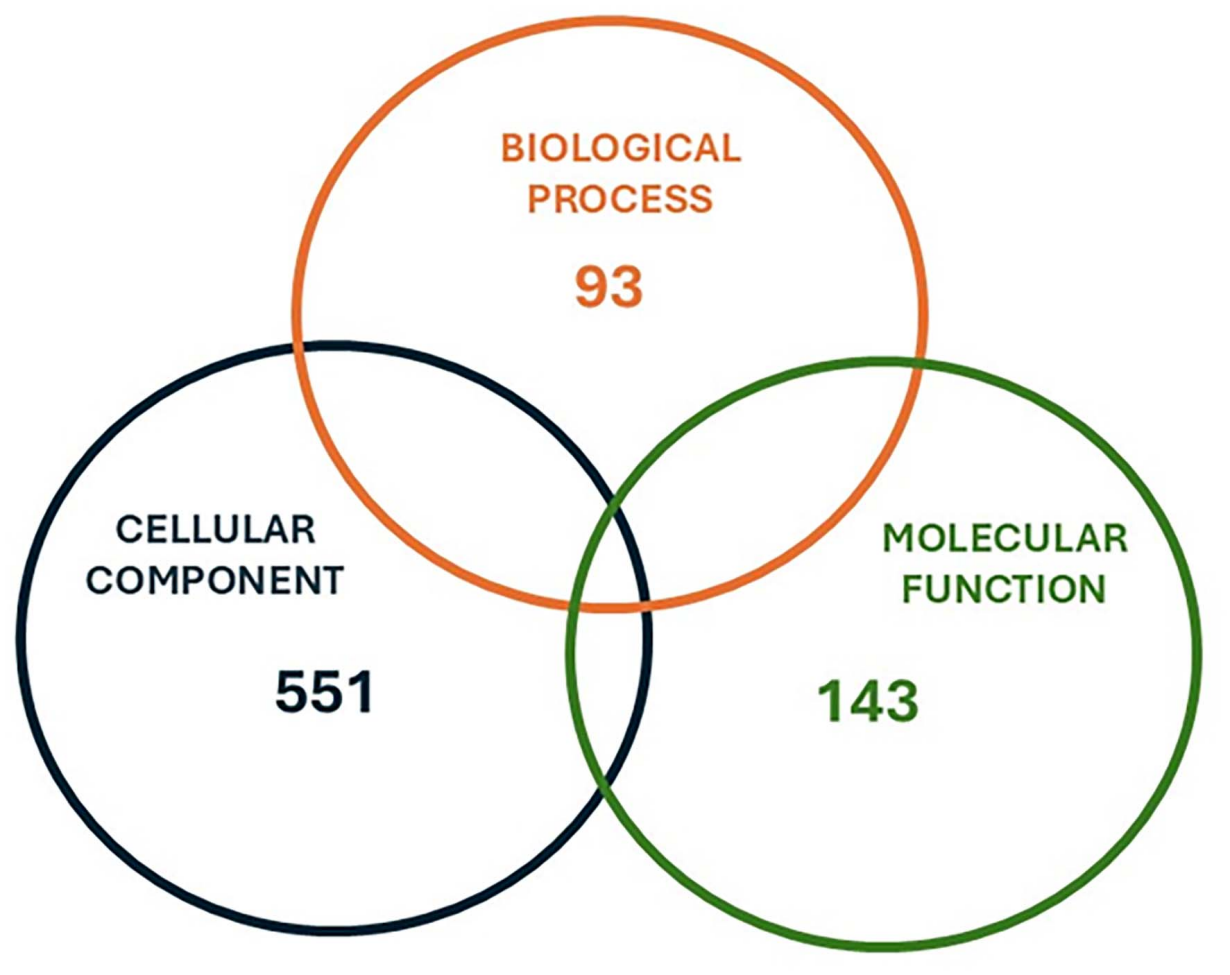
Venn diagram showing the distribution of total identified proteins related to bone metabolism.

When evaluating the enriched BPs involved in the detected proteins, a correlation with bone metabolism can be observed (figure 3). Proteins related to ossification, bone development, skeletal system development, bone mineralization and osteoblast differentiation correspond to a total of 93 identified genes.

**Figure 3.**
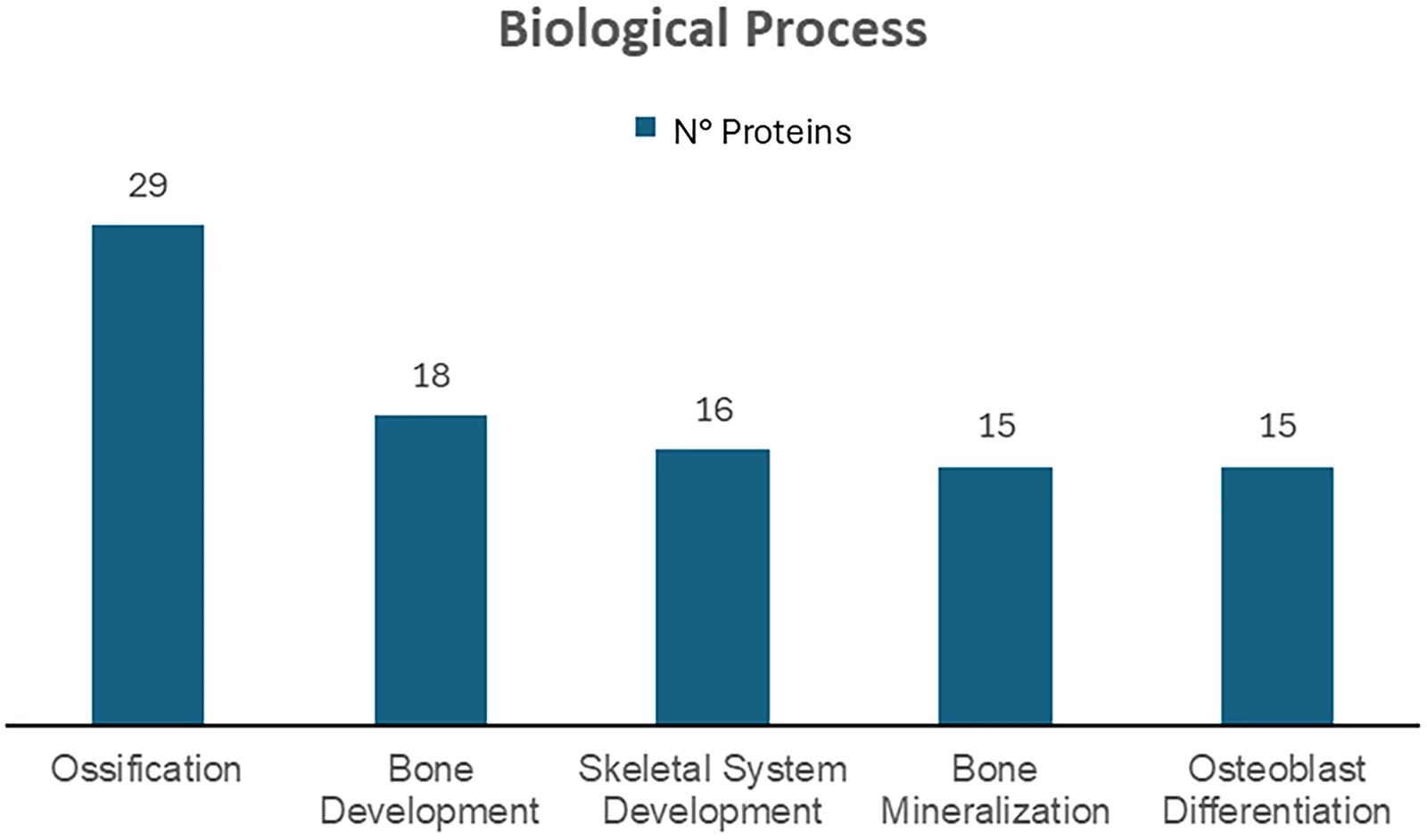
Bar chart of biological processes involving bone metabolism.

The most enriched MFs highlight proteins related to calcium ion binding, collagen and calcium-binding proteins, totalling 143 genes. These findings reinforce the involvement of these proteins in pathways directly associated with bone metabolism.

The enriched CCs mainly point to structures associated with bone tissue metabolism, with a focus on collagen in the extracellular matrix, the extracellular matrix, the basement membrane and different types of collagen, including collagen types I, V and IX. These elements represent fundamental components of bone matrix architecture and remodelling, especially collagen type I, which constitutes the majority of the organic bone matrix. By integrating these findings, it is observed that 551 genes are directly related to bone tissue.

Results from the KEGG for differentially abundant proteins and the number of proteins associated with each KEGG pathway term were found using the DAVID software (figure 4). Pathways related to bone metabolism stand out, such as ECM-receptor interaction, parathyroid hormone synthesis, secretion and action, PI3K–Akt signalling pathway, Hedgehog signalling pathway, Wnt signalling pathway, totalling 116 genes identified in these metabolic pathways.

**Figure 4.**
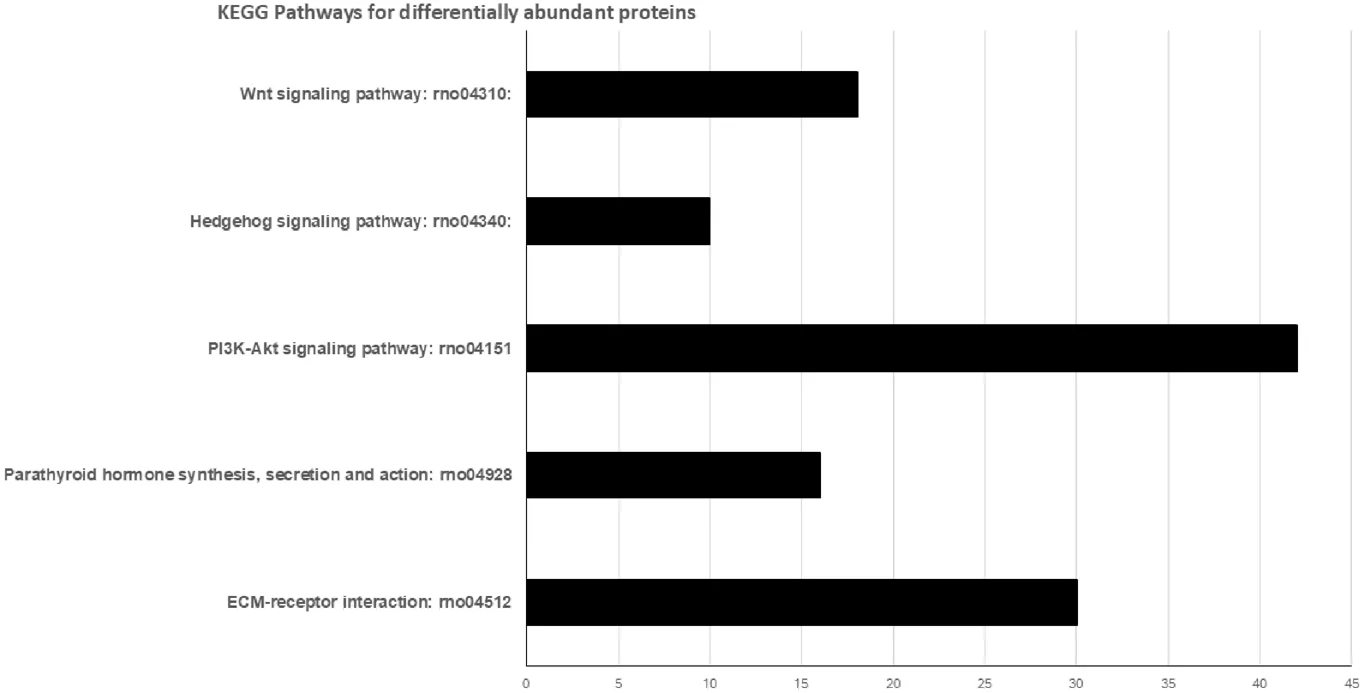
KEGG pathways for differentially abundant proteins related to bone metabolism.

### 3.2. Key proteins identified

Representative proteins were selected to illustrate the major functional classes of the bone extracellular matrix, including structural components, mineralization-related proteins, regulators of bone remodelling and signalling molecules.

By using the protocol described, several proteins strongly related to bone tissue were identified. The protein collagen type 1 alpha 1 chain (COL1A1) is described in the literature as causing osteogenesis imperfecta when there is a mutation in the formation of this protein [16]. Mutations in this gene disrupt collagen synthesis and fibril organization, leading to reduced bone mineral density, impaired mineralization, decreased bone strength and increased fracture susceptibility. COL1A1 encodes the α1 chain of type I collagen, the most abundant protein in bone and the principal organic component of the bone extracellular matrix. During bone resorption, osteoclast-mediated degradation of type I collagen generates multiple COL1A1-derived peptide fragments, many of which can be detected in serum and urine and correlate with established biochemical markers of bone turnover [17,18].

The results of the study by Wang *et al.* [19] demonstrated that the C-type lectin domain family 11 member A (Clec11a) can bind to integrin alpha-11 protein and promote the differentiation of mesenchymal cells into mature osteoblasts, revealing its importance in bone metabolism. Yue *et al.* [20] showed that this protein is related to the maintenance of the adult skeleton, as it is responsible for osteogenesis. This was observed in both their *in vitro* and *in vivo* studies, where low expression of this protein resulted in reduced bone volume.

Secreted protein acidic and cysteine rich (SPARC) is a non-collagen protein expressed in mineral tissue, also referred to as osteonectin or basement membrane protein. The SPARC protein contains the domain for collagen and hydroxyapatite binding. Rats with depletion of this protein exhibit altered phenotypes such as short tail, lower bone mineral density and smaller femur size. It is expressed by osteoblasts and plays a role in their regulation. SPARC is related to bone remodelling and maintenance of bone mass and quality [21].

The study by Shainer *et al.* [22] demonstrated that biglycan (Bgn) is a protein found in the extracellular matrix and is present in bone and cartilage. It is related to bone formation, and the authors discuss in their work that depletion of this protein leads to alterations in bone formation and bone regeneration.

Regarding the role of histones, specifically the role of histone H4, Adithya *et al.* [23] sought to understand the epigenetic modifications of histones during osteoblastic differentiation and evaluated that histones can undergo post-translational modifications such as acetylation, methylation and phosphorylation that lead to modifications in signalling pathways for osteoblastic expression. The authors suggest that histone modifications help us understand cellular regulation and differentiation at the chromatin level and evaluate in this review that histone can lead to activation or suppression of osteogenesis. The results indicate that with the use of technology, histones can be modified and promote the differentiation of stem cells into osteogenic lineage cells for the treatment of bone abnormalities.

Among the other proteins found in the samples of the present study, the extracellular matrix phosphoglycoprotein (Mepe) is known to be involved in the regulation of a series of metabolic processes in the body, including regulation of renal phosphate excretion (inhibitory action); regulation of bone mineralization by osteoblasts and cartilage mineralization by chondrocytes; and regulation of mineralization of the extracellular matrix of the craniofacial complex (e.g. teeth, bones and cartilage) [24–26]. It is also said to be able to increase the proliferation of dental pulp stem cells.

Another protein also present in the results of the present study was integrin-binding sialoprotein (Ibsp). Ibsp, also known as bone sialoprotein 2 (BSP2), is a glycoprotein mainly found in bone tissue [27,28]. High blood concentrations of the protein have been observed in various cancer cases [27,29,30], and studies indicate that it plays an important role in tumour growth, invasion and metastasis by promoting angiogenesis, cell migration and adhesion in an immunologically favourable microenvironment [27,31,32]. The literature also reports that bone sialoprotein is a key factor in the regulation of mineralized tissue formation, and that gene ablation of Ibsp in mice results in impaired bone mineralization and growth, as well as defective osteoclastogenesis [33]. Regarding the craniofacial region, the effects of gene ablation include reduced acellular cementum formation, hypomineralization of alveolar bone and detachment of periodontal ligaments, resulting in periodontal collapse [33].

In addition to the Mepe and Ibsp genes, the samples in the present experiment also had different types of collagen, containing different chains. One of the identified chains was Col6a1, also known as collagen type VI alpha 1 chain. The literature suggests that mutation or loss of collagen type VI is linked to a range of musculoskeletal disorders, with a strong relationship to muscular dystrophies, fibrosis and/or tissue ossification, and hip osteoarthritis [34]. In the study conducted by Christensen *et al.* [34], they evaluated the function of collagen type VI in the morphology and physical properties of bone and cartilage tissue in the knee joint of Col6a1(−/−) mice. Based on the results obtained, the authors observed that Col6a1(−/−) mice showed lower trabecular bone volume, lower connective density, fewer trabeculae and lower trabecular thickness compared to Col6a1(+/+) animals [34].

Another collagen of notable relevance observed in the present study is encoded by the COL2A1 gene, also known as collagen type II alpha 1 chain. This, in turn, is the most predominant collagen in articular cartilages synthesized by chondrocytes [35]. Mutations in the respective gene are responsible for causing a series of skeletal dysplasias, ranging from Stickler syndrome and Kniest dysplasia to congenital [35–38]. The study by Rolvien *et al.* [35] demonstrated that hemizygous loss of the COL2A1gene in mice can influence skeletal architecture, significantly impacting cortical and trabecular bone mass.

In addition to the collagen-coding genes, the presence of the gene encoding collagenase-3 (MMP13), also known as matrix metalloproteinase 13, has been identified. Regarding the possible functions of the respective protein, Ponte *et al.* [39] suggest that MMP13 derived from mesenchymal cells may regulate the number and/or activity of osteoclasts, as well as bone resorption and mass. The authors suggest that, according to the observed results, deletion of the MMP13 gene caused a 50% reduction in the number and surface of osteoclasts, increasing cortical bone volume [39]. Additionally, Tomczyk-Warunek *et al.* [40] indicate collagenase-3 as responsible for bone and cartilage resorption through the degradation of collagen fibres (mainly type II) found in the matrix of these tissues.

### 3.3. Unexpected and contaminant proteins

A small proportion of proteins not typically associated with bone tissue was detected. Protein characteristic of blood and soft tissues, such as haemoglobins and actins, accounted for less than 1.8% of the identified proteins, indicating that the initial removal of surrounding tissues was effective. In addition, a limited number of unexpected proteins were identified at very low abundance. Such identifications are recognized as an inherent limitation of MS-based proteomics and may result from trace exogenous contamination, co-purification or analytical carryover during sample preparation and LC–MS/MS analysis [41,42]. Given their negligible abundance, these proteins were not considered to influence the overall proteomic profile or the biological interpretation of the results.

### 3.4. Network analysis (STRING/confidence cut-off)

PPI analysis was performed using the STRING database with a high-confidence interaction score (0.700), and disconnected nodes were excluded from the network. The resulting network comprised 1468 proteins and revealed a highly interconnected molecular landscape (figure 5). Owing to the complexity of the complete network, an enlarged view of the principal extracellular matrix (ECM) interaction module is presented to facilitate biological interpretation (figure 6).

**Figure 5.**
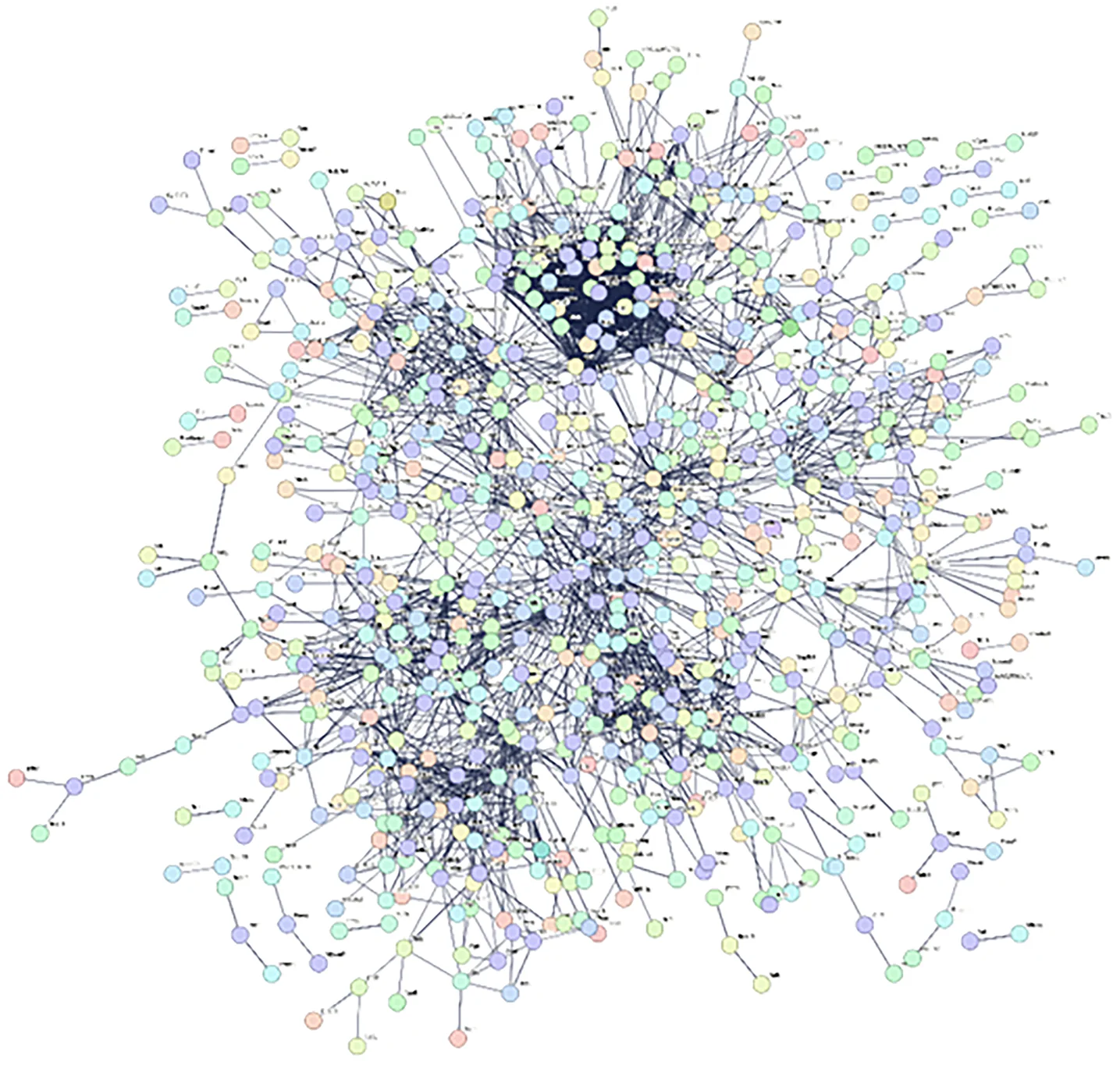
Protein–protein interaction network.

**Figure 6.**
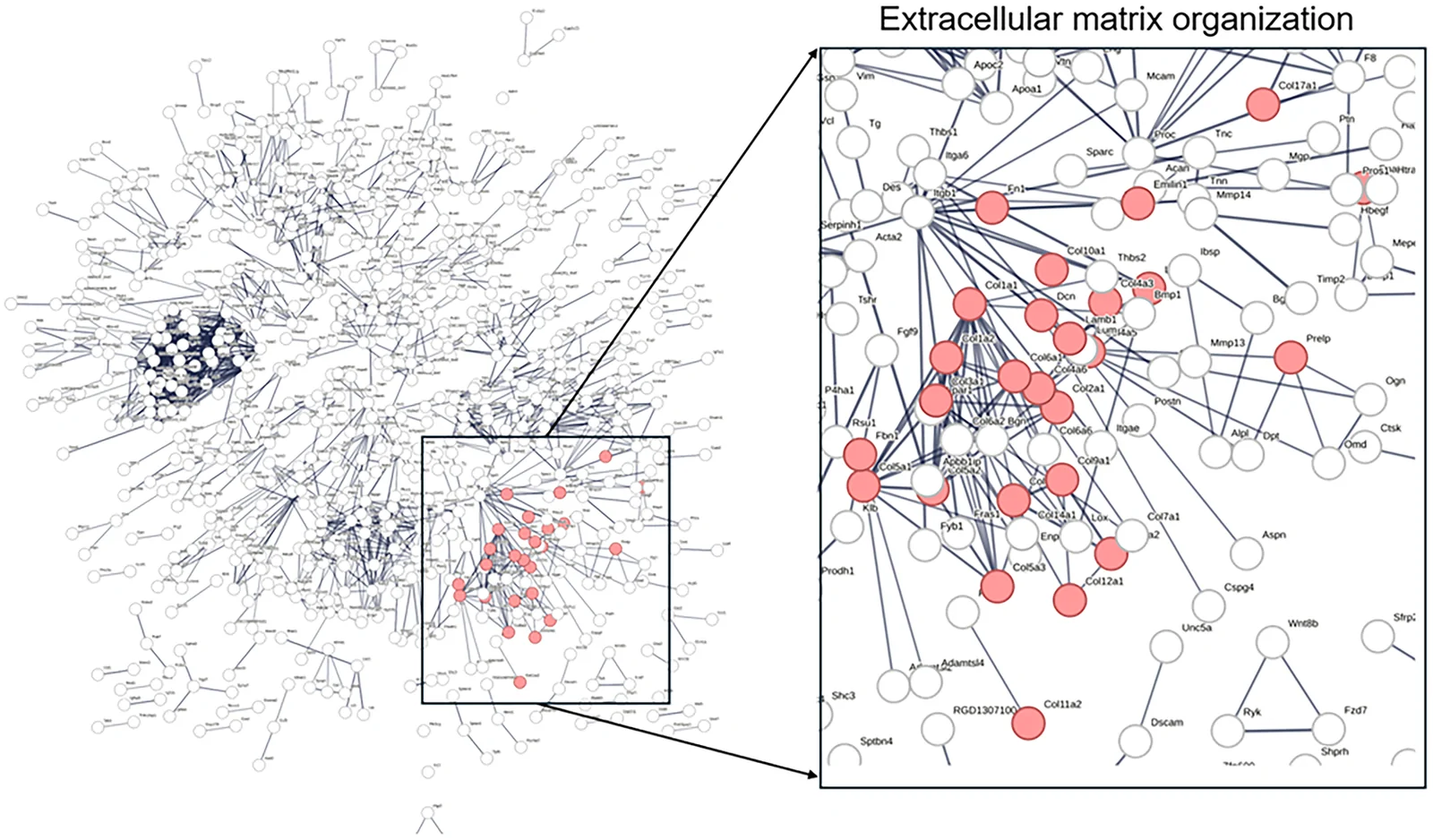
Protein–protein interaction network of the identified bone proteome generated using the STRING database. The right panel shows an enlarged view of the principal extracellular matrix (ECM) cluster, highlighting representative proteins involved in bone matrix organization, collagen assembly, mineralization and bone remodelling. Type I collagen proteins (COL1A1 and COL1A2) occupy a central position within the network and are connected to other major bone ECM proteins, including SPARC, IBSP, BGN, DCN, POSTN, FN1, MMP13, TIMP2, BGLAP and BMP1, demonstrating the extensive interactions among structural and regulatory components of the bone extracellular matrix.

To further investigate molecular pathways associated with bone formation, proteins annotated with the GO BPs bone mineralization, bone morphogenesis, bone development and ossification were analysed separately using STRING (figure 7). This analysis identified a densely connected interaction module predominantly composed of extracellular matrix proteins and osteogenic regulators. Core proteins, including COL1A1, COL1A2, COL3A1, COL5A1, SPP1, POSTN, LAMB3, MMP14 and TIMP2, occupied central positions within the network and displayed extensive interactions with other bone-related proteins.

**Figure 7.**
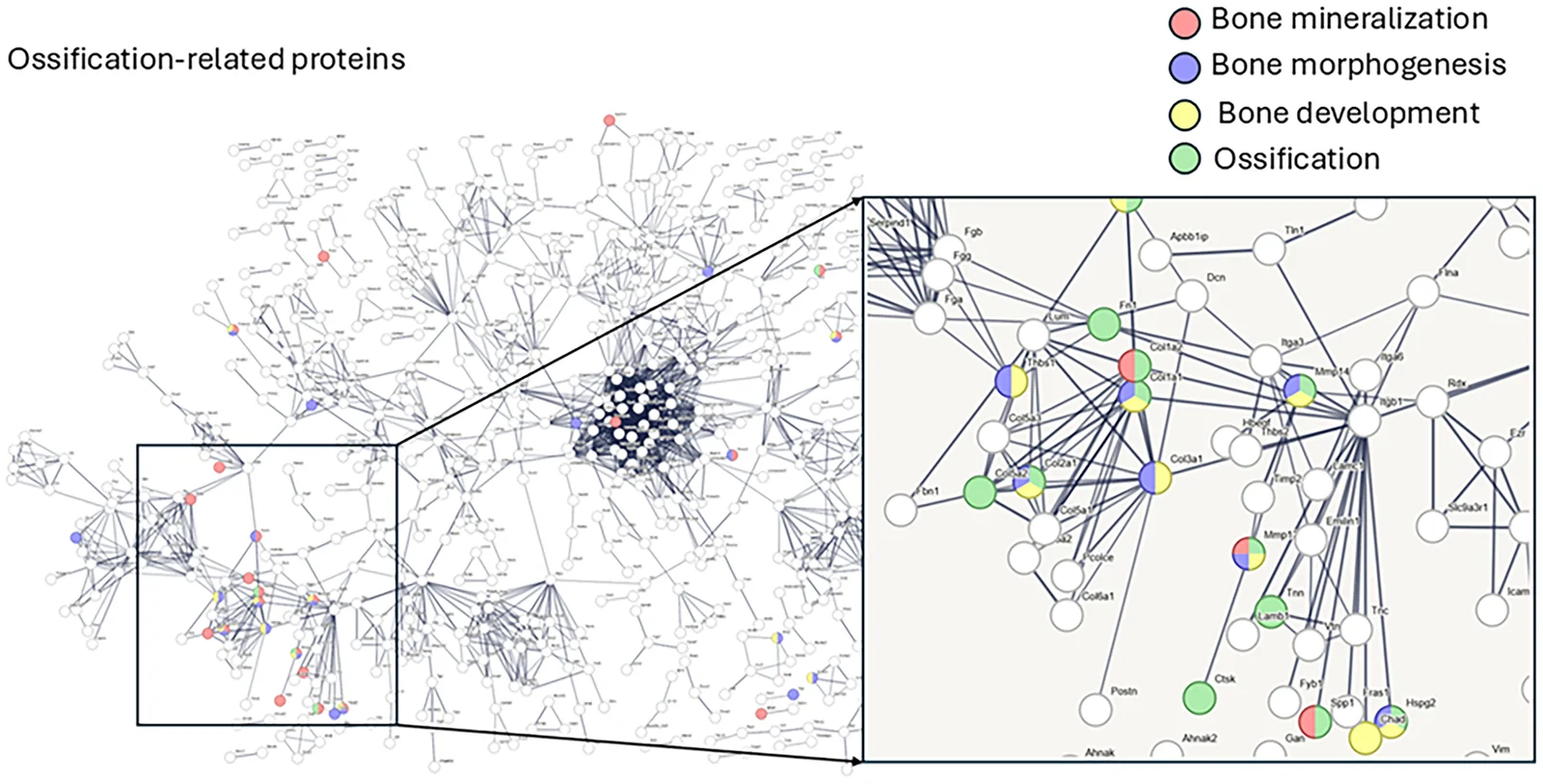
Protein–protein interaction (PPI) network of ossification-related proteins generated using the STRING database. Coloured nodes indicate proteins associated with the Gene Ontology (GO) biological processes of bone mineralization (red), bone morphogenesis (purple), bone development (yellow) and ossification (green), while white nodes represent interacting proteins added by the STRING analysis.

## 4. Discussion

The extraction of proteins present in bone tissue samples continues to be a major challenge for researchers from various fields [11], mainly owing to high mineralization, low cellularity and strong association of proteins with the mineralized extracellular matrix. While the literature presents a wide variety of well-established protocols for protein extraction from soft tissues, both of animal and human origin [43–45], there is a significant gap in standardized and reproducible methodologies for bone tissue. Despite bone tissue being one of the most abundant tissues in the human body, few studies describe in detail and systematically the necessary steps for efficient protein extraction. In this context, this study contributes by providing a detailed and standardized protein extraction workflow for bone tissue proteomics, demonstrating its successful application across the analysed small bone samples and facilitating methodological reproducibility in future studies.

The present study was methodologically based on the article by Jiang *et al.* [13] published in 2007, which represents a relevant contribution to the field by presenting different approaches for protein extraction from bone tissue, including protocols with and without demineralization. Although the study provides important methodological guidance, some aspects related to the description of critical steps, such as the use of inhibitors, incubation times, temperatures, sample storage conditions and other experimental parameters, were not described. This limitation is understandable considering the methodological context at the time, but it may hinder the full reproducibility of the protocol, especially for research groups starting proteomic studies in hard tissues. In this sense, the present work seeks to complement and expand the previously proposed methodology, offering a more detailed and standardized description of these steps.

The results demonstrate that the methodology used for protein extraction from bone tissue was effective, highlighting that 1429 known proteins were identified for the species *R. norvegicus* and 138 unknown proteins. Jiang *et al.* [13] reported in their article that approximately 1429 proteins were successfully identified, and upon evaluating the results of the study in question, we can see that by following the proposed protocol, we obtained the same amount of proteins described by the authors.

The analysis identified numerous proteins with well-established roles in bone extracellular matrix organization, mineralization and bone remodelling, including biglycan, periostin, osteocalcin, S100 proteins and SPARC, among others.

This demonstrates that proteomic analysis allows for the evaluation of changes in bone metabolism, including resorption, neoformation and remodelling, by observing whether certain proteins are more or less expressed according to their biological function.

When analysing the results of signalling pathways, the identification of different pathways closely related to bone metabolism is observed, with a particular emphasis on the Wnt and Hedgehog (IHH) pathways. These pathways are directly associated with the maturation and activity of osteoblasts, as well as playing fundamental roles in the development of different organs. The Wnt pathway influences the proliferation and differentiation of mesenchymal stem cells, progenitor cells of osteoblasts, osteoclasts and bone homoeostasis. On the other hand, the IHH pathway helps maintain bone homoeostasis. Studies in mice with IHH gene depletion show the absence of osteoblasts during endochondral ossification, although the formation of osteoblasts through intramembranous ossification is still observed. IHH gene signalling is necessary for perichondrial cells to initiate osteoblast differentiation [46–48].

This work is not limited to demonstrating a method for protein extraction but proposes the standardization of a protocol applicable to future experiments, with clear technical details. In this way, it aims to help address the scarcity observed in recent literature regarding well-established protocols for protein extraction from bone tissues, providing researchers with a clear, reliable methodological description that can be applied in future studies.

It is important to acknowledge some limitations of the proposed protocol. The sequential extraction procedure is labour-intensive and requires considerable processing time, as well as specialized reagents, which may limit its routine application. Furthermore, low-abundance proteins may not be fully recovered, which could result in selective losses during the extraction process. Another limitation of the present study is that the demineralization solution and the initial extraction buffer were not reanalysed, since these fractions had already been characterized in the original sequential extraction protocol described by Jiang *et al.* [13]. This study focused on refining and expanding this established workflow by incorporating an additional extraction step and standardizing the extraction procedure. Future studies should investigate the proteomic composition of each extraction fraction to further optimize protein recovery—particularly for mineral-associated proteins—and to provide a more comprehensive understanding of protein distribution throughout the extraction process. Despite these limitations, the proposed protocol has the potential to be adapted to different experimental contexts, including different species and bone types, taking into account variations in bone density, mineralization and tissue architecture.

## Supplementary Material



## Data Availability

The data produced during this research have been archived in the Zenodo repository [49]. Supplementary material is available online [50].
